# Exploring dose–response variability and relative severity assessment in STZ-induced diabetes male NSG mice

**DOI:** 10.1038/s41598-024-67490-z

**Published:** 2024-07-17

**Authors:** Steven R. Talbot, Miriam Heider, Martin Wirth, Anne Jörns, Ortwin Naujok

**Affiliations:** 1 Institute of Clinical Biochemistry, Hannover Medical School, 30625 Hannover, Germany; 2 Hannover Medical School, Institute for Laboratory Animal Science, Carl-Neuberg-Straβe 1, 30625 Hannover, Germany

**Keywords:** NSG mice, Diabetes, Streptozotocin, Severity assessment, RELSA, Diabetes, Carbohydrates

## Abstract

NSG mice are among the most immunodeficient mouse model being used in various scientific branches. In diabetelogical research diabetic NSG mice are an important asset as a xenotransplantation model for human pancreatic islets or pluripotent stem cell-derived islets. The treatment with the beta cell toxin streptozotocin is the standard procedure for triggering a chemically induced diabetes. Surprisingly, little data has been published about the reproducibility, stress and animal suffering in these NSG mice during diabetes induction. The 3R rules, however, are a constant reminder that existing methods can be further refined to minimize suffering. In this pilot study the dose–response relationship of STZ in male NSG mice was investigated and additionally animal suffering was charted by applying the novel ‘Relative Severity Assessment’ algorithm. By this we successfully explored an STZ dose that reliably induced diabetes while reduced stress and pain to the animals to a minimum using evidence-based and objective parameters rather than criteria that might be influenced by human bias.

## Introduction

The NOD.Cg-Prkdc^scid^ Il2rg^tm1Wjl^/SzJ mouse, commonly called NSG mouse, is a versatile, immunodeficient model abundantly used in laboratory animal science. Their almost complete absence of immune cells makes them invaluable for xenotransplantation, including the engraftment of human hematopoietic stem cells, peripheral blood mononuclear cells, and cancer cells^1–3^. In the field of experimental diabetes mellitus (DM), diabetic NSG mice are frequently employed to assess the functionality and phenotype of primary human pancreatic islets^4^ or human stem cell-derived pancreatic organoids^5^ after subcutaneous injection or transplantation under the kidney capsule. To induce diabetes in these animals, streptozotocin (STZ), a highly selective beta cell toxin, is widely used either through a single-dose or multiple low-dose injection regimen^6^. STZ offers the advantage of rapid, selective beta cell mass destruction by necrosis within 24–48 h, resulting in absolute insulin deficiency, which, in turn, leads to weight loss due to the depletion of fat depots and muscle atrophy^7,8^. However, these experiments' reproducibility, uniformity, and appropriateness of STZ dosage for efficacy and animal welfare are critical considerations^7^. Administering doses that are too high may increase mortality, while doses that are too low may not reliably induce diabetes and render experimental data from these animals useless^9^. Mortality can be caused by direct organ STZ toxicity (pancreas, liver, kidney), DM-related complications, or severe hypoglycemia caused by massive beta-cell loss via necrosis within the first 24 h after STZ injection^7,10^. Some protocols estimate a 20% loss of animals due to severe hypoglycemia caused by massive beta-cell necrosis^9^ and even higher mortality due to DM-related complications^11^. In addition, STZ is famous for its species-, strain-, sex- and inter-lab-specific effects on rodents^7^. Unfortunately, data on the responsiveness of laboratory mice exposed to different concentrations of STZ are scarcely published and typically do not show the ratio of responding to non-responding animals. Also, the severity is seldom published or contextualized with the administered doses of STZ.

In accordance with animal welfare regulations enforced by national authorities, a weight loss exceeding 20% is a typical threshold for a humane endpoint, necessitating euthanization of the animals^12,13^. However, due to insulin's specific roles in glucose and fatty acid metabolism, weight loss may occur without exhibiting significant distress^14^. Furthermore, if experimental milestones, such as sufficient connection of islet grafts to the animal's bloodstream, have not been achieved by the time of the 20% weight-loss threshold, euthanization may occur without yielding any experimental benefit. Consequently, using body weight as a sole marker may be less precise and should be supplemented with other objective parameters^14^.

**Figure 1:**
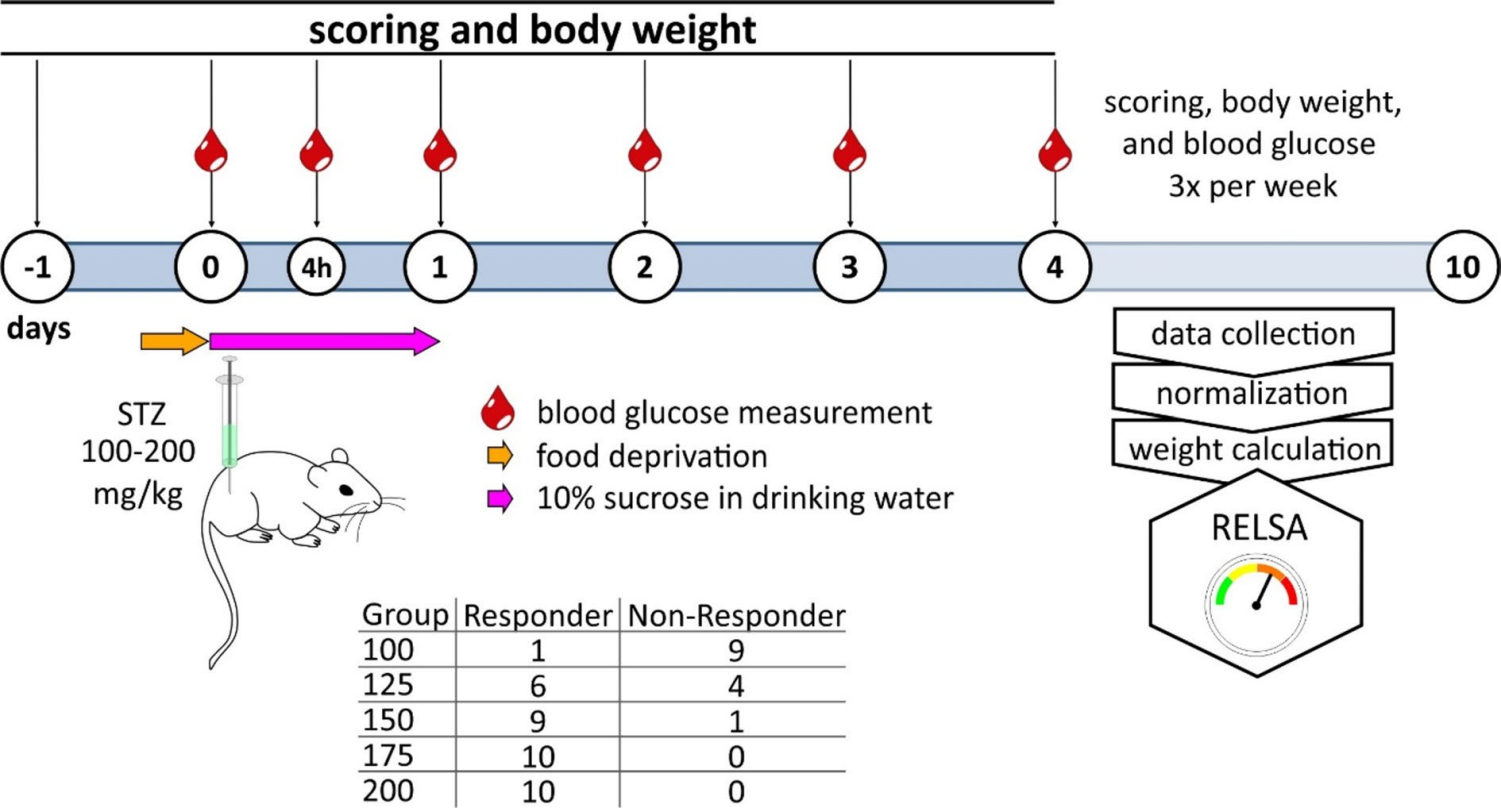
Schematic presentation of the experimental proceedings.

In this pilot study, we aimed to investigate the dose–response relationship of STZ in male NSG mice with two primary objectives. First, we sought to determine an STZ dose that reliably induces DM while limiting animal suffering. Second, we applied the novel ‘Relative Severity Assessment’ algorithm (RELSA)^15^ to assess animal welfare based on the easily measurable and objective parameters of body weight and blood glucose concentration. Our study successfully identified an STZ dose that consistently induced DM while causing only a moderate weight loss of approximately 10% within the observation period. Notably, the application of RELSA revealed that individual parameters for body scoring of diabetic animals may be insufficient to represent animal welfare objectively. Furthermore, we will elucidate the methodology and present the findings of our study, emphasizing the results of our inquiry into the distinction between responders and non-responders among NSG mice exposed to varying doses of STZ treatment (Fig. 1).

## Results

### Dose-dependent effect of STZ on blood glucose levels in NSG mice

**Figure 2:**
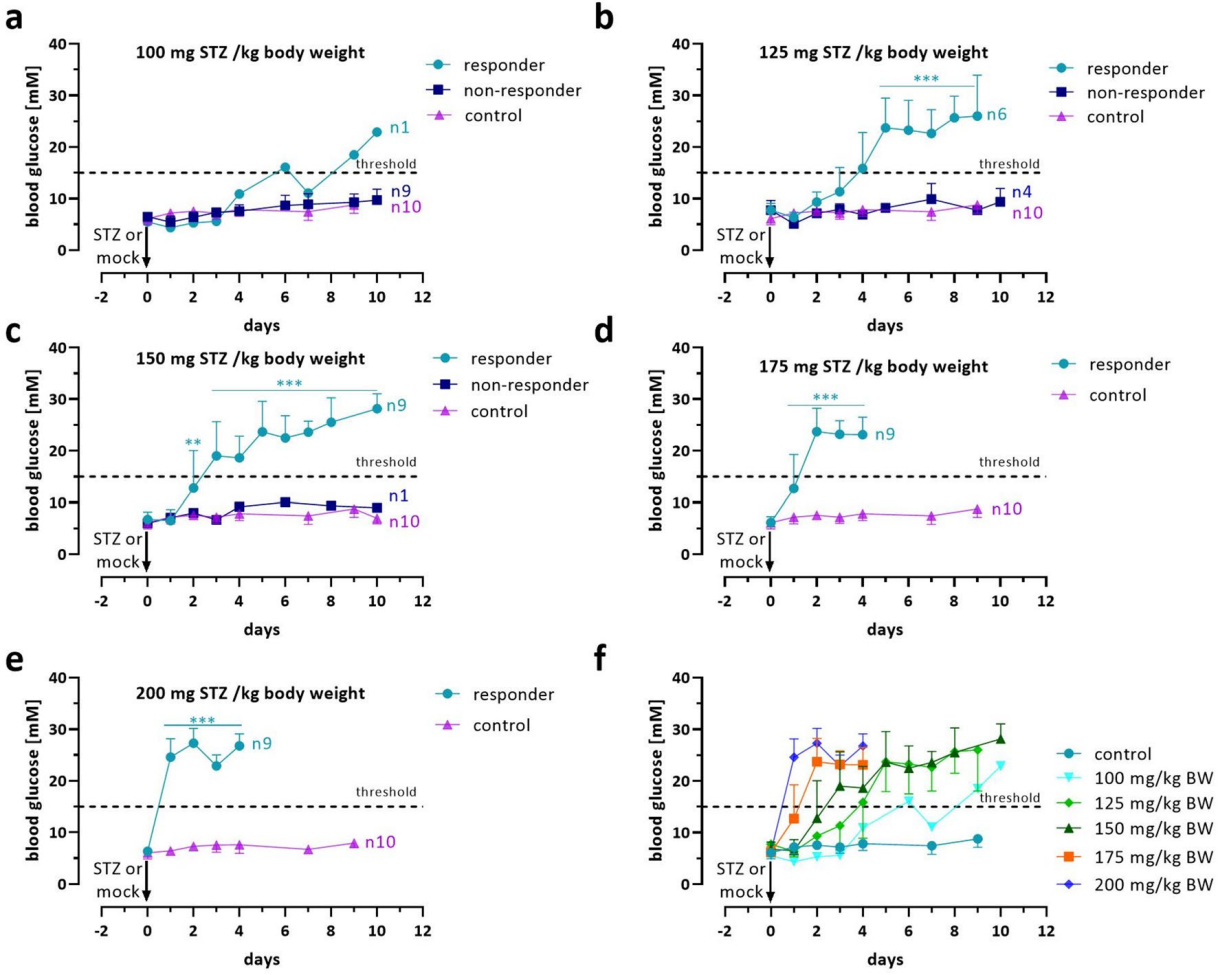
Dose-dependent effect of STZ on blood glucose levels in NSG mice. Changes in blood glucose values in mmol/l over 9–10 days of STZ-treated NSG mice are shown vs. mock controls. The mice were injected i.p. with 100 (**a**), 125 (**b**), 150 (**c**), 175 (**d**), or 200 mg STZ per kg body weight (**e**). Separate curves are presented for diabetic (responders) and non-diabetic animals (non-responders). Data are presented as means ± SD, n = 9–10. The groups were compared on individual days with *Student’s t*-test, corrected for multiple comparisons by the Bonferroni–Dunn method, resulting in adjusted p-values: ***p ≤ 0.001, **p ≤ 0.01, *p ≤ 0.05. In (**f**), a summary of the blood glucose changes of all diabetic animals (responders) is presented. The dotted line marks the threshold value of 90%, which indicates successful diabetes induction after two consecutive blood glucose measurements above 15 mmol/l.

**Figure 3:**
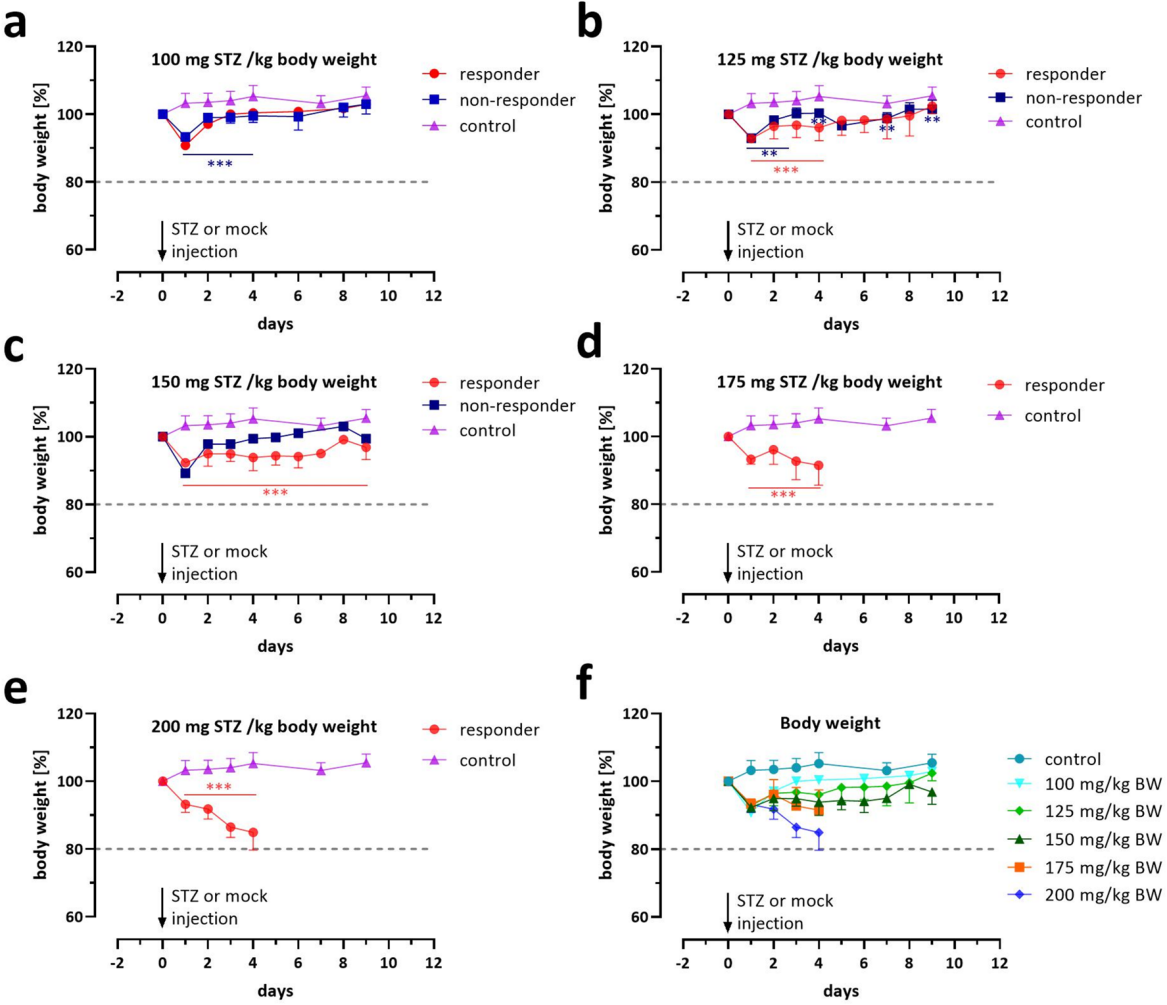
Dose-dependent effect of STZ on the body weight of NSG mice. Shown are changes in body weight percentage to the baseline weight calculated at day 0 over 9–10 days of STZ-treated NSG mice vs. mock controls. The mice were injected i.p. with 100 (**a**), 125 (**b**), 150 (**c**), 175 (**d**), and 200 mg STZ per kg body weight (**e**). Separate curves are presented for diabetic (responders) and non-diabetic animals (non-responders). Data are presented as means ± SD, n = 9–10. The groups were compared on individual days with *Student’s t*-test, corrected for multiple comparisons by the Bonferroni-Dunn method, resulting in adjusted p-values: ***p ≤ 0.001, **p ≤ 0.01, *p ≤ 0.05. In (**f**), a summary of the changes in body weights for all diabetic animals (responder) is presented. The dotted line marks the 20% weight loss threshold value that indicates a body condition score that requires euthanasia.

Streptozotocin (STZ) is an antibiotic that induces pancreatic islet β-cell destruction in rodents after it has been taken up by the cells via the GLUT2 glucose transporter. Two immediate symptoms of this beta cell destruction are hyperglycemia and weight loss (Figs. 2 and 3). In NOD.Cg-Prkdc^scid^ Il2rg^tm1Wjl^/SzJ (NSG) mice, single-dose i.p. injection of different STZ concentrations ranging from 100 to 200 mg STZ per kg body weight yielded a reliable chemical induction of diabetes in three out of five concentrations (Fig. 2a–e). 100 mg STZ was not sufficient to induce diabetes in 9 out of 10 NSG mice, and the single responding mouse showed a delayed blood glucose increase with a fully diabetic state reached as late as day 10 of the observation period. The STZ concentration of 125 mg led to diabetes manifestation in 6 of 10 animals, which, according to our threshold parameters, could only be classified as chemically induced diabetes from day 4–6 onwards for most NSG mice except one (diabetes manifestation at day 10).

**Figure 4:**
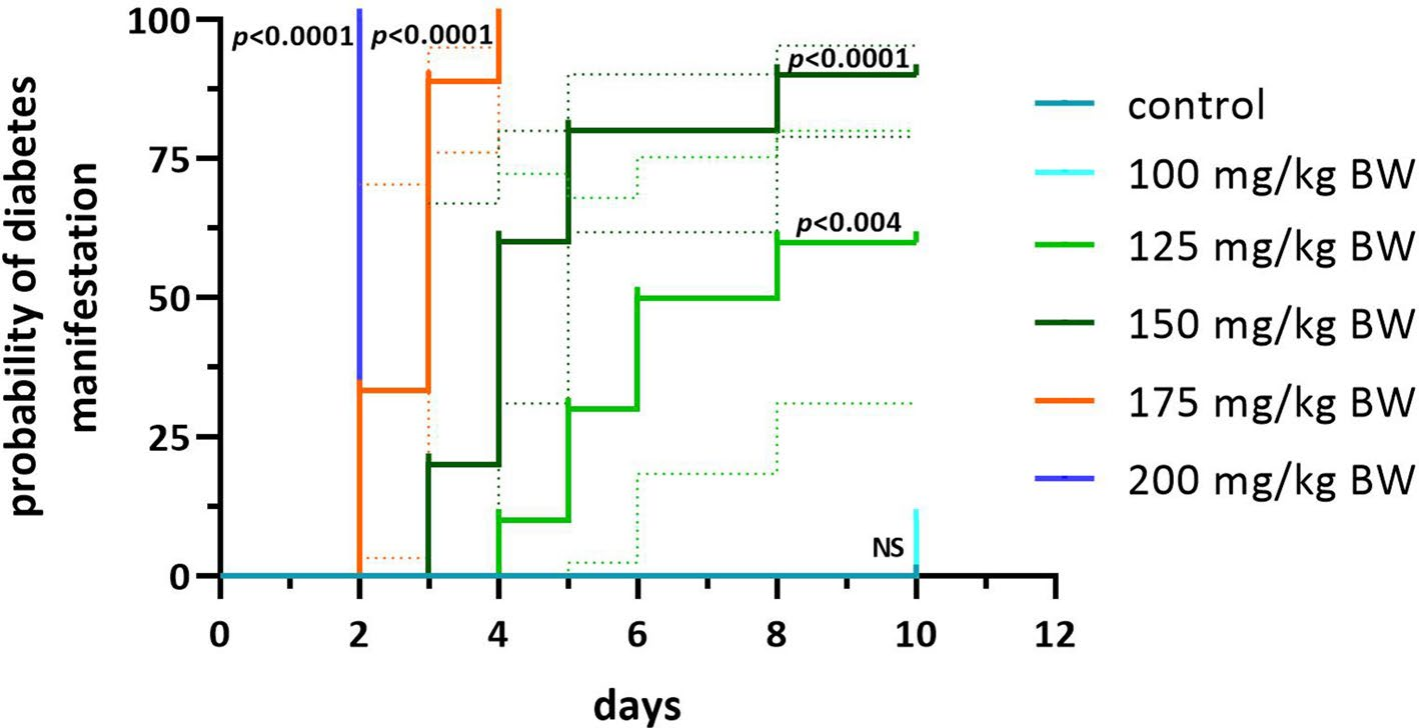
Kaplan–Meier survival analysis of STZ-treated NSG mice. The probability of diabetes manifestation in NSG mice after treatment with STZ ranged from 100 to 200 mg STZ per kg body weight compared to the mock control (in teal) over the time course of the study. Statistical differences were calculated using the Log-rank (Mantel-Cox) test. The dotted lines represent the 95% confidence intervals.

For the 150 mg dose, 9 out of 10 animals were diagnosed with diabetes, which typically became apparent between days 3 and 4. The other NSG mice developed stable hyperglycemia on days 5 and 8. The two high STZ concentrations, however, led to a fulminant and complete manifestation of diabetes in all animals. At 175 mg, most animals developed diabetes on days 2–3 of the observation period, and one animal only converted on day 4. At 200 mg, all animals were uniformly diagnosed as diabetic on day 2. Figure 2f depicts the summary of blood glucose curves for all responding animals and shows the correlation between the STZ concentration used and the dynamics of diabetes development. Next, a mixed-effects hierarchical analysis of variance (ANOVA) was used to estimate the effect of STZ treatment and time (days) on blood glucose (Supplementary Table 1). A highly significant effect for the dose variable was found (F(5, 38) = 122.5, p < 0.0001), indicating that different doses resulted in significantly different blood glucose concentrations. Further, the main effect of time was also significant (F(8, 38) = 11.2, p < 0.0001), suggesting differences between days of blood glucose measurement. There were also significant differences between responding and non-responding animals (F(1, 38) = 50.9, p < 0.0001), indicating contributions to the variance in glucose levels. Finally, the interaction between treatment and day was significant (F(4, 38) = 3.8, p = 0.011), indicating that the effect of treatment on blood glucose levels depended on the day of measurement and that the impact of dosing varied over time. The interaction between treatment and responding mice was insignificant (F(1, 38) = 0.4, p = 0.512), showing that the response was not directly dose-dependent. The within-subjects by time error analysis also showed a highly significant effect ((F(38, 337) = 15.9, p < 0.0001)), indicating substantial within-subject variability across different days that was explained by the treatment-by-day interaction. An equally large error effect could be observed in the day:responder interaction ((F(11, 337) = 14.6, p < 2e-16) showing substantial variation across days between responder and non-responder mice. The time animals responded to the STZ treatment was, therefore, different. However, the three-way dose:day:responder error term interaction was not significant ((F(14, 337) = 1.0, p = 0.42)), indicating that the two other interaction terms already explained most of the error sources. Overall, the efficacy of the STZ doses varied over time and showed strong individual bias, highlighting the importance of considering both factors, STZ dose and time, in the model.

Diabetes caused by an absolute insulin deficiency typically leads to severe weight loss due to lipolysis of fatty tissue and muscle atrophy over time. All NSG mice in this experiment treated with STZ showed an immediate weight loss of 7.1 ± 0.5% (SD) percent within 24 h of the injection, irrespective of the STZ concentration, except for the mock-injected controls that gained approximately 3% weight (Fig. 3a–e). In groups 100 and 125 mg STZ, we observed a linear weight gain after the first 24 h so that all but one of the animals could almost reach or exceed their initial weight within the experiment (Fig. 3a,b). The body weight curves for diabetic and non-diabetic animals were very similar overall. At 150 mg, the diabetic mice showed a slight increase in body weight after the initial 24 h loss. Still, at the end of the experiment, the body weight was significantly reduced compared to the control but stabilized without further loss (Fig. 3c). The two high STZ concentrations, however, led to a fulminant and continuous loss of body weight in all animals, predominantly at 200 mg STZ (93.1% ± 4.0 and 84.9% ± 5.3 [SD], mean body weight loss compared to the baseline) within 96 h after STZ injection (Fig. 3d,e). Figure 3f presents a summary of all body weight curves for all responding animals. The hierarchical analysis of variance (ANOVA) was also used to estimate the effect of STZ dose and time (day) on body weight (Supplementary Table 2). A highly significant effect for the dose variable was found (F(5, 40) = 41.9, p < 0.0001)), indicating that different doses resulted in significantly different body weight changes. Further, the main effect of time was also significant (F(7, 40) = 4.5, p < 0.001), suggesting differences between days of measurement. There were no significant differences between responding and non-responding animals (F(1, 40) = 0.005, p < 0.942). Finally, the interaction between treatment and day was significant (F(4, 40) = 3.1, p = 0.039), indicating that the effect of dose on body weight change values depended on the day of measurement and that the impact of dosing varied over time. The interaction between dose and responding animals was insignificant (F(1, 40) = 0.14, p = 0.707), showing that the response was not directly dose-dependent.

The within-subjects by time error analysis also showed a highly significant effect (F(38, 316) = 10.13, p < 0.0001), indicating substantial within-subjects variability across different days that was explained by the treatment-by-day interaction. No differences in the interaction terms for the errors of time:responder (F(10, 316) = 1.23, p = 0.274) and dose:time:responder (17,3316) = 1.4, p = 0.134) were found. Overall, the efficacy of the doses varied over time and showed strong individual bias that was not fully explained with the included variables.

**Figure 5:**
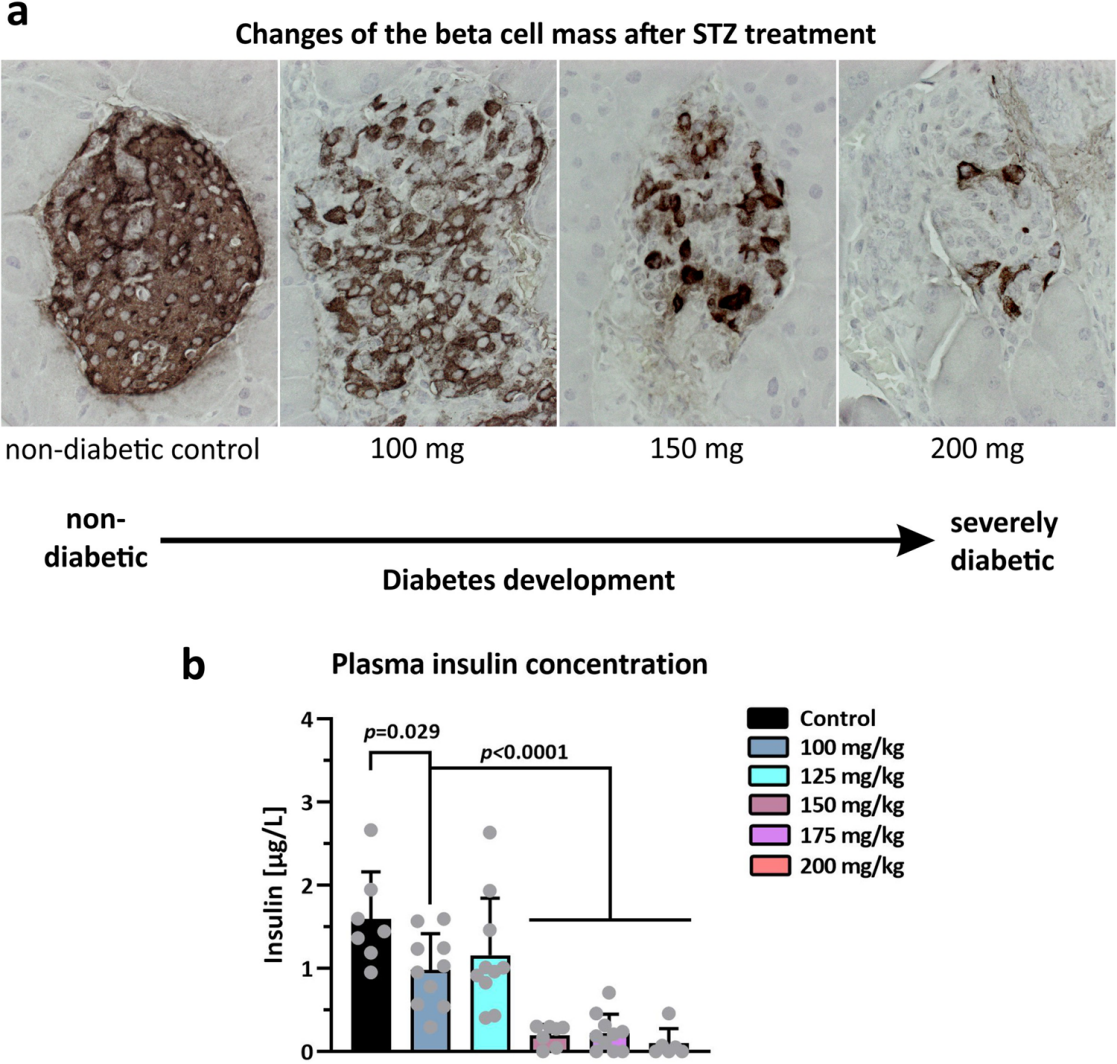
Immunohistochemical analysis of insulin. (**a**) Shown is an immunohistochemical DAB-staining of insulin on pancreatic slices obtained from mock-treated NSG mice, NSG mice with 100 mg STZ/kg body weight, NSG mice with 150 mg STZ/kg body weight, and (**c**) NSG mice with 200 mg STZ/kg body weight (images from left to right). Scale bar = 50 µM. ×40 magnification. (**b**) Plasma insulin concentrations in µg/L of control mice and STZ-treated mice on the day of their sacrifice as means ± SD. Statistical differences were calculated using ANOVA plus *Dunnett’s* post hoc test. P-values are depicted above each group. Data points from seven animals measured below the detection limit of 30 pg/ml are indicated as zero in the diagram.

The survival analysis summarizes the response of NSG mice to different STZ concentrations over time (Fig. 4). A rapid and reliable diabetes induction was observed for the 175 and 200 mg groups. In comparison, at 150 mg, high blood glucose levels occurred with a significant delay in nine out of ten mice, whereas 125 and 100 mg STZ per kg body weight proved unreliable (Fig. 4). In the controls, small, medium, and large islets were well preserved and, in the islets, pancreatic beta cells depicted a dense insulin immunostaining (Fig. 5a).

After the treatment with 100 mg STZ, the number of islets per pancreatic sections as well as the beta cells in the islets were moderately reduced to around 50%. In addition, the insulin immunostaining in the beta cells was fainter compared to those of the normoglycemic (non-diabetic) controls. The beta cell loss after 150 mg STZ treatment was enormously increased so that only around a quarter of islets were present with a small number of remaining insulin-positive cells. After the highest concentration of 200 mg STZ, only some islets remained detectable with only some insulin-positive beta cells (Fig. 5a). To substantiate the loss of the beta cells shown in the immunohistochemistry data, we measured the plasma insulin concentration in mice on the day of their sacrifice (Fig. 5b). In control mice we measured 1.42 ± 0.29 µg/L insulin (mean ± SD, range 0.952–1.948). Mice treated with 100 and 125 mg STZ showed a slightly reduced insulin concentration. The insulin concentration value after 150 mg STZ injection was 0.20 ± 0.12 (mean ± SD, range 0–0.30) thereby imposing a loss of more than 80% plasma insulin compared to the control mice. Thus higher concentrations than 125 mg caused a massive and highly significant drop of plasma insulin with seven animals showing data below the detection limit of the ultrasensitive ELISA (1 for 150 mg, and each 3 for 175 and 200 mg) (Fig. 5b).

### Dose and time-dependent RELSA trajectories

**Figure 6:**
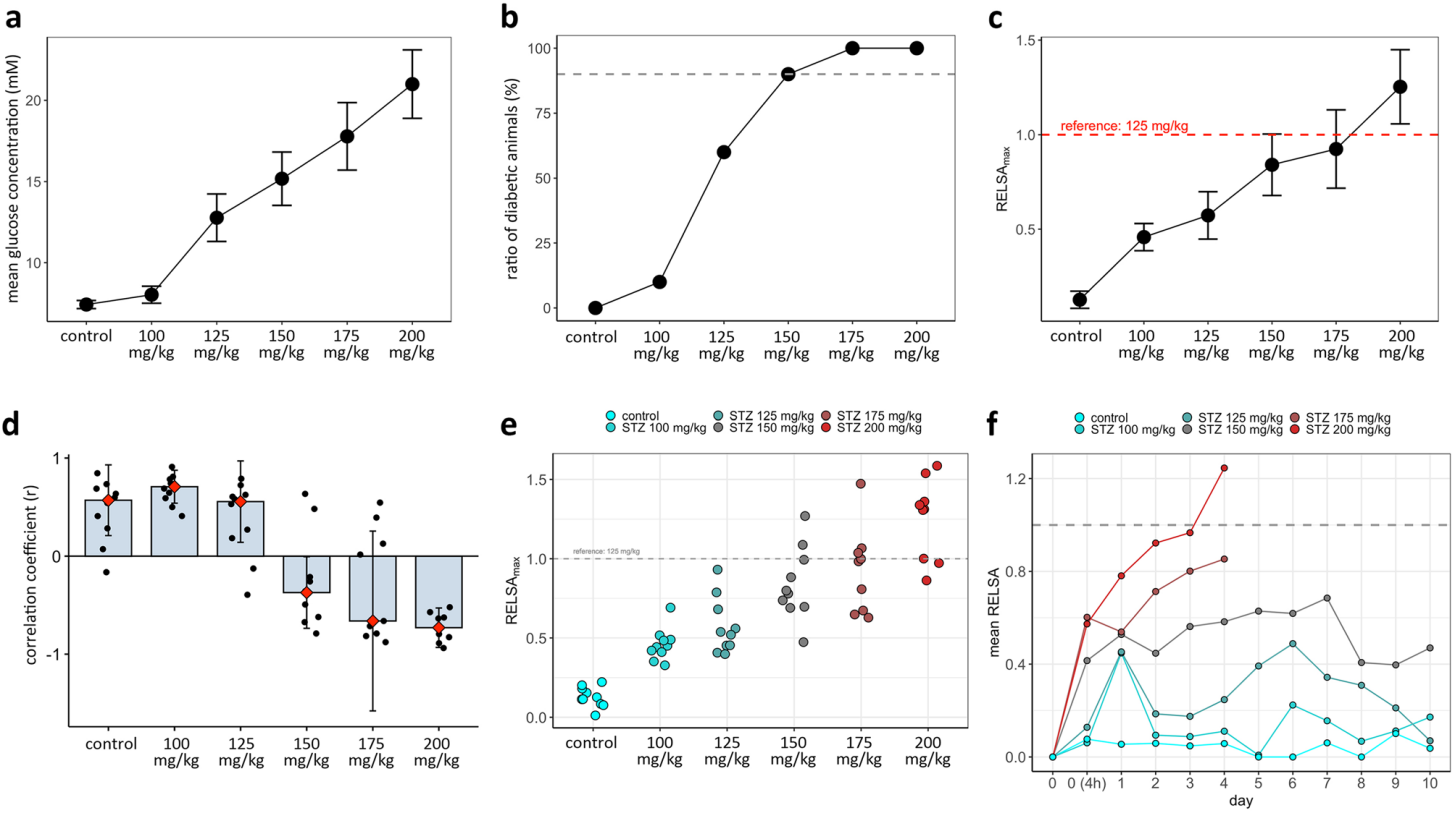
RELSA calculations to measure the severity of diabetes induction by STZ. (**a**) Time-independent glucose concentration in STZ-treated and control mice. (**b**) The ratio (%) of diabetic animals to non-diabetic animals at each STZ concentration. The dotted line marks the 90% success rate for diabetes induction. (**c**) The RELSA_max_ value is the maximum reached RELSA score in each animal and tested dose. The averaged RELSA_max_ values show a linear increase to higher severity with higher doses. Subsequent post hoc tests indicated significant differences between treated and mock-injected mice. ANOVA F(5,48) = 31.5 p ≤ 0.004 for 100 mg STZ, p ≤ 0.0001 for 125, 150, 175 and 200 mg STZ. (**d**) A zero-lag cross-correlation coefficient of body weight and blood glucose time series for each dose is shown. Depicted are medians (red rhombs) and error bars (IQR). Data points represent individual data of each mouse in mock-injected mice and all treatment groups. Until 125 mg/kg STZ, the trajectories of glucose and body weight show a positive correlation over time. This changes at 150 mg/kg STZ, when glucose levels increase, and body weight loss occurs. (**e**) Cluster analysis and severity categorization of the six STZ-treated and control mice groups. Here, the individual RELSA_max_ data are shown. The between-subjects variance also increases with higher doses. (**f**) Time-resolved mean RELSA curves for STZ-treated and control mice. RELSA data were averaged on each day and dose. The dashed gray line at RELSA = 1 indicates the threshold of the reference set (125 mg/kg STZ). Values exceeding the threshold had on average, worse values than the reference set. All analyzed animals were mapped relative to that position.

Figure 6a depicts the mean blood glucose per STZ dose. The curve follows a sigmoidal curve and shows that for each unit in glucose (mM), the Odds Ratio in favor of the diabetic event was 1.44 (CI_95%_[1.35; 1.58]). The analysis of the ratio of NSG mice with successfully induced diabetes also revealed a sigmoidal curve. In this pilot study, we assumed an arbitrary 90% threshold for conversion success. Thus, only successfully converted animals that had become diabetic were eligible for subsequent experiments. At this threshold, we observed the 150 mg STZ dose as the minimum required dose for achieving this goal (Fig. 6b).

Finally, the RELSA algorithm was applied to determine the suffering of the animals based on the two measured objective parameters, changes in blood glucose and body weight for each dose and day (Fig. 6c–f). We also calculated the cross-correlation between the changes in body weight and blood glucose time series (Fig. 6d). The mean maximum severity of the diabetes-inducing treatment of each group is presented in Fig. 6c, and the individual maximum stress of the animals is shown the scatter plot in Fig. 6f. A likelihood-ratio test compared the full model against the Null model, containing only the IDs as random effects. The IDs explained 37.41% of the total variance information (X^2^ = 622.48, df = 53, p ≤ 0.0001). Therefore, we included the IDs in the dose and time-dependent analyses.

The ANOVA table of the regression (Supplementary Table 3) showed significant effects for dose, day, and the interaction of dose and day. Each change in STZ doses was associated with a significant increase in the RELSA value. In the RELSA_max_ data, we found a significant between-dose effect (F(5, 52) = 7.42, F = 39.16, p ≤ 0.0001). The post hoc analyses showed a significant result when comparing control vs. 100 mg STZ (p ≤ 0.001) and 150 mg STZ (p ≤ 0.0001).

The time series of body weight and blood glucose showed a positive cross-correlation at low STZ doses and a negative at the highest dose (Fig. 6d). At 150 and 175 mg STZ, this correlation was inconclusive, with some animals in each group gaining weight and some losing despite elevated blood glucose (Fig. 6d). Therefore, the average RELSA response depends on changes in blood glucose concentration and body weight.

However, analysis of the mean RELSA values over time showed a stabilization at 100 mg and 125 mg STZ as values were closer to the RELSA reference level of 1. However, the trend shows that the RELSA values of the 150 mg STZ group plateaued at 0.54 ± 0.11 SD, while the 175 and 200 mg groups indicated a steep incline in values, partly exceeding 1.

## Discussion

Laboratory animals in science, particularly mice, play a unique and important role in behavioral and biomedical research in basic and preclinical studies. Although many scientific questions can be answered today using in vitro tissue culture assays or other alternative methods, such as computer-assisted predictions, laboratory animals cannot yet be entirely dispensed for scientific purposes. The NSG mouse is a versatile model used in various fields, e.g. immunology, oncology, and experimental diabetology. It can be efficiently humanized by engrafting human cells and tissues without acute xenograft immune rejection. In DM research, NSG mice, prior made diabetic by the selective β-cell toxins alloxan or streptozotocin^6^, are commonly used as a recipients for human β-cells, either stemming from primary islets, insulinoma cell lines, or β-cells derived from pluripotent stem cells by in vitro differentiation^5,11^. As such, they are the preferred tool for evaluating antidiabetic cell therapy approaches and serve as a crucial predictor of the efficacy of such therapies for translation purposes later in humans. The DM caused by STZ results in an absolute insulin deficiency and induces considerable weight loss and animal suffering. This is caused by the lack of the antilipolytic effect of insulin on fatty tissue and by proteolysis in the skeletal muscles, which suffer from a lack of energy^16^. As animal experiments are naturally subjected to moral and legal restrictions, animal welfare must be strictly monitored with a considerable emphasis on body weight^17,18^.

Insulin injections might counteract moderate weight loss, but a loss of more than 20% is typically considered a threshold that requires no intervention but termination of the experiment^12,13^. These measures are necessary to stay within the legal limits of national laws. However, research indicates that assessing welfare based on single parameters like weight loss in a setting of absolute insulin deficiency might be less suited^14^.

Secondly, such insulin supplementation may not be in line with the actual aims of the experiment, for example, when evaluating the therapeutic potential of transplanted human β-cells which might not have yet fully engrafted and have connected to the animal’s blood circulation via angiogenesis at that time. The aim of this pilot study was, therefore, to define a single dose of STZ that, on the one hand, reliably triggers DM in NSG mice and, at the same time, reduces animal suffering to the lowest possible level. Secondly, we report the distribution of animals responding or non-responding to a given STZ dose. Despite a large number of publications on the subject (> 35,000 entries on PubMed, as of March 2024), researchers are permanently obliged to refine the methods (3R) used within an animal experiment in such a way that the most excellent possible welfare is achieved for the animals^19^. Thus, to objectively stress-test the scoring system, data on body weight and blood glucose were used to analyze animal welfare and stress using the new RELSA procedure over the time course of the experiment^15^. Data from these two parameters are quick and easy to collect, causing very little stress to the animal. A well-defined STZ dose would then allow the animals to be kept for longer in the experiment, maximize animal welfare, and improve the achievability of the objectives of a typical transplantation experiment. To the best of our knowledge, although STZ has been used for decades, such a systematic approach precisely for male NSG mice using i.p. STZ injections has not yet been published. Importantly, in our study protocol, STZ was immediately injected after dissolution as recommended^9,20^. Therefore, the effects we observed are mainly attributable to the activity of the α-anomer of STZ (see method section)^21,22^. The anomer composition of STZ is typically subjected to mutarotational changes into a 50:50 distribution of α- and β-anomers within the first 30–60 min after dissolution in acidified buffer^21^. STZ β-anomers have been shown to have a reduced efficacy^22^ in exchange for a possibly reduced mortality^21^. To minimise variability during experimentation, great care should be taken to adhere strictly to a predetermined study protocol, either by immediate injection of STZ or by using equilibrated STZ solutions.

Of the five concentrations tested, only 150, 175, and 200 mg STZ/kg body weight produced reproducible hyperglycemia and therefore diabetes. This aligns with other STZ studies in the NSG mouse model^23^ or other mice strains^24–26^. 100 mg STZ proved to be unsuitable, and at 125 mg, only a low penetrance of 60% was observed. The presence of a high number of beta cells and islets at 100 mg STZ clearly showed that this concentration is rather unsuitable. 150 mg STZ was comparatively more effective: The induced DM showed a slowly progressive course with manifestations between days 3 and 8 of the study. Here, the average body weight loss was only about 6% over a total observation period of 10 days, and the immunohistochemical staining showed a reduced but still detectable beta cell mass. Hence, the risk of ketoacidosis appears unlikely due to the residual beta cell function. This will probably contribute significantly to reducing mortality and morbidity in longer-term investigations. Nonetheless, the required severity of diabetes with blood glucose values well above 22 mmol/L is close enough to the human situation and thus well-suited for islet transplantation experiments. In contrast, the two high STZ concentrations showed a fulminant course of blood glucose increase, with an equally fulminant increase of severity, which, judging from the kinetics of the body weight and RELSA curves, would soon require euthanasia.

The dose–response curve we selected showed a linear relationship between the mean blood glucose concentration and the STZ dose. However, a plateau is expected at even higher doses of STZ. High STZ concentrations are often associated with hypoglycemia-induced mortality in the immediate hours after STZ injection^27^ and increased morbidity and mortality in long-term experiments^7^. No animal died because of hypoglycemia. Likewise, no animals were lost during the 10-day experimentation period, although two animals, one treated with 200 mg STZ and one with 175 mg STZ, scored 4 on the last day of the study. Strikingly, the evidence-based RELSA analyses showed that 175 and 200 mg STZ were only minimally more effective in causing diabetes but caused a significantly higher burden due to the increase in blood glucose levels and the concomitant loss of body weight. Time-resolved mean RELSA curves confirmed increased animal suffering over time, especially at high STZ concentrations. Notably, looking at individual physical parameters is not necessarily meaningful when assessing the welfare of diabetic animals, as shown here by the correlation of body weight gain/loss with blood glucose gain/loss. The assessment of the general condition, fur status, activity, and alertness of animals may also harbor the risk of bias during subjective scoring. Parameters that are easy and accurate to measure, such as body weight and blood glucose used in this study, further substantiated in the future by parallel assessment of body temperature, activity (monitored in a spinning wheel), ketone bodies in urine, and water consumption bear the advantage that they can be objectively measured and evaluated. Data entry into the RELSA application on handheld devices directly inside the animal facility would then provide a timely calculation of animal burden.

This study was performed on male mice. Female mice and rats are known for lower vulnerability to STZ than males. The lower sensitivity can be slightly or very pronounced^28,29^. This variability will most likely also result in a different severity profile. The lower sensitivity has been partly attributed to the female sex hormone estradiol and its corresponding receptor pathway, which protects female mice from STZ toxicity^30^. However, in light of the differences in outcomes of long-standing diabetes between female and male human patients, appropriate models are required to adequately address the issue of gender-associated diseases in type 1 and type 2 diabetes^31^. In the future, further efforts should be made to develop reliable protocols for diabetes induction in female mice while minimizing animal suffering.

## Conclusions

In summary, we can show that a single dose of 150 mg STZ per kg body weight can reliably induce DM in male NSG mice. Higher concentrations were not profoundly more effective but produced needlessly more animal suffering, as shown by the RELSA data using two input parameters that are easy to assess directly within the animal facility. The 3R rules are a constant reminder that even seemingly established procedures can be further refined to reduce the suffering of laboratory animals. We conclude that the RELSA algorithm is ideally suited to assess the many facets of animal welfare in the diabetic NSG mouse model.

## Methods

### Housing

Housing and experimentation of laboratory animals in this study were conducted in accordance with the ARRIVE guidelines^32,33^. NOD.Cg-Prkdc^scid^ Il2rg^tm1Wjl^/SzJ (NSG) mice (JAX stock #005557) were housed in groups sized two to four in an air-conditioned specific pathogen-free room at 21 °C and 50% humidity, following a 12:12 h light/dark cycle, in individual cages within ventilated cabinets (Scanbur, Karlslunde, Denmark) equipped with filter bonnets. These cages were furnished with autoclaved softwood granulate (poplar wood, AB 368P, AsBe-wood GmbH, Buxtehude, Germany) as nesting material, along with autoclaved cotton rolls (ANT Tierhaltungsbedarf, Buxtehude, Germany) and enrichments (igloo, gnawing material). The mice had unrestricted access to drinking water and a gamma-irradiated (25 kGy) standard breeding diet (Altromin TPF-1324, Lage, Germany). Food was removed from the cages for 4 h during fasting periods while drinking water remained accessible. All human interactions, including handling, weighing, cage maintenance, i.p. STZ injections, blood glucose measurements, and scorings were carried out under a laminar flow utilizing sterile tools to ensure aseptic conditions.

### STZ injection and diabetes induction

Male mice aged 8–12 weeks were utilized in this study. The experimental groups were allocated randomly by technical staff, not involved in the planning and experimental steps of the study. The animals' baseline starting weight at the experiment's beginning was 27.6 g ± 2.1 (SD, range 23.3–32.1 g). STZ injections were prepared by dissolving STZ (Santa Cruz Biotechnology, U-9889, Dallas, Teas, USA; α-anomer content 88.78%) in various concentrations (10–20 mg/ml) in sodium citrate acidified PBS, pH 4.5, and were promptly administered within 5 min. To induce diabetes, STZ was injected intraperitoneal (i.p.) to male mice that had fasted for 4 h, with a dosage ranging from 100 to 200 mg of STZ per kg of body weight. To ensure precise administration of the STZ dose and injection volume, the animals' body weights were measured the day before the experiment and immediately before the STZ injection.

Blood glucose levels were monitored using tail-tip blood samples, both before and 4 h after STZ injection, utilizing a Contour Next glucometer (Ascensia Diabetes Care, Basel, Switzerland). To mitigate hypoglycemia-induced morbidity and mortality, the mice had their drinking water replaced with a sterile-filtered 10% sucrose solution for up to 24 h. A diagnosis of diabetes was established when blood glucose levels exceeded 15 mmol/L in two consecutive measurements on different days. The study spanned 9–10 days, as depicted in Fig. 1 of the study design.

### Animal scoring

Diabetic and non-diabetic animals were euthanized no later than 10 days after the initiation of the experimental protocol. Following the detection of diabetes, the animals underwent bi-daily scorings, which included daily assessments of both body weight and blood glucose levels. A specific table for the body scoring system was used, including the general appearance, facial expression, body posture, activity, behavior, and polyuria/polydipsia (Supplementary Table 4). Non-diabetic animals were scored thrice weekly, with concurrent blood glucose and body weight measurements. In instances of rapid and severe disease progression, particularly at elevated STZ concentrations, animals were euthanized no later than 96 h after STZ injection or 96 h after the manifestation of diabetes.

To ensure animal welfare and compliance with German regulatory requirements, two consecutive blood glucose measurements exceeding 30 mmol/L were considered as a criterion for study termination, owing to the potential risks associated with life-threatening hypovolemia and/or ketoacidosis. A second criterion for discontinuation, in accordance with German authorities' guidelines, was a loss of over 20% of initial body weight.

Euthanasia was conducted by administering a CO_2_ overdose at a 25% fill rate, followed by subsequent exsanguination through cardiac puncture. Plasma was isolated from the blood samples and frozen at − 80 °C for later determination of the insulin concentration. The pancreases were then extracted for subsequent histological and immunohistochemical analyses.

### Immunohistochemistry and insulin ELISA

Pancreatic sections from 4% paraformaldehyde-fixed tissue, embedded in paraffin, of the different STZ-treated groups and the control group were stained either with the avidin–biotin-complex or double immunofluorescence technique with primary antibodies for all islets cells, especially beta cells as well as for glucagon and delta cells. After the removal of the paraffin and after overnight incubation with the first antibody, biotinylated goat anti-rabbit or anti-guinea pig Ig G (1:200; 30 min) and a streptavidin–biotin–peroxidase complex (1:1000; 30 min) (both from Jackson Immuno Research, West Grove, IL, U.S.A.) were used as second antibodies. The peroxidase was demonstrated with 0.7 mM diaminobenzidine and 0.002% H_2_O_2_ in 0.05 mM Tris HCl buffer, pH 7.6. Analyses were performed using an Olympus BX61 microscope. Plasma insulin concentrations were determined using an ultrasensitive mouse insulin ELISA following the manufacturer’s instructions (Mercodia, Uppsala, Sweden).

### RELSA analysis

Calculations were conducted in the R software (v4.3.1)^34^, using RELSA, ggplot2, and dplyr packages. The RELSA procedure was applied, as outlined by Talbot et al.^15^, to estimate the relative positions of individual animals within an abstract severity space. The input data comprised the group, dose, and time information, as well as the animals' glucose and body weight change data. The input data were normalized based on each animal's initial data point. The 125 mg/kg group was used as the reference in the RELSA calculations to establish relative severity context. This reference defined the highest levels of impairment to animal welfare across all input variables within the RELSA space, normalized to RELSA = 1.

Further, the RELSA procedure fuses the multidimensional normalized input data of glucose and body weight change into a single scalar for relative severity comparisons. Consequently, this allowed for the relative positioning of the analyzed animals in relation to the reference. When animals showed values above RELSA = 1, this corresponded to larger escalations in the input variables than in the reference set. The individual RELSA values were used to generate averaged and time-dependent RELSA trajectories. Additionally, a RELSA_max_ analysis was employed to identify time-independent maximum severity values in each animal, facilitating the identification of group-specific severity or extreme values.

### Statistics

Statistical analyses of individual parameters presented in Figs. 2, 3, and 4 were carried out using the GraphPad Prism analysis software (GraphPad, San Diego, CA, USA). The unpaired *Student's t*-test was employed and corrected for multiple comparisons using the Bonferroni-Dunn method, resulting in adjusted p-values denoted as *** for p ≤ 0.001, ** for p ≤ 0.01, and * for p ≤ 0.05. Additionally, the data were analyzed using a time-dependent hierarchical ANOVA (see supplemental Table 1 and 2). The log-rank (Mantel-Cox) test was applied to evaluate diabetes manifestation rates compared to the control. Detailed p-values are presented in the legend of Fig. 4.

RELSA results were analyzed with a linear mixed-effects regression (lmer) to estimate the time and dose-dependent effects and their interaction, using the lme4 and lmerTest R-packages. The animal ID was integrated as a random effect to account for the within-subjects correlation. It is important to note that the analysis excluded data from the 4-h time point on day 0. The significance of the animal ID was assessed using a likelihood-ratio test against the Null model. The coefficient table was subsequently transformed into an ANOVA table, enhancing its readability by utilizing Satterthwaite's method to approximate the degrees of freedom in the presence of interactions.

For the analysis of time-independent RELSA_max_ results, a linear model was employed. Post hoc tests were used to estimate between-dose contrasts, with adjustments made using the Tukey procedure to mitigate family-wise errors. Results were reported as an ANOVA table and post hoc contrasts.

Furthermore, we explored zero-lag cross-correlation of the individual time series of body weight change and blood glucose levels per STZ dose administered. The individual coefficients were averaged and plotted, exploring the average parameter dominance per STZ dose.

### Ethics approval

All animal experimentations performed in this study were in line with the German Animal Welfare Act regulations and the approval granted by the Lower Saxony State Office for Consumer Protection and Food Safety (AZ 33.12-42502-04-21/3793).

## Supplementary Information

Supplementary Information.

## Data Availability

Raw data used and/or analyzed in this study can be obtained from the corresponding author upon request.
