# Author Correction: Exploring dose–response variability and relative severity assessment in STZ-induced diabetes male NSG mice

**DOI:** 10.1038/s41598-025-97524-z

**Published:** 2025-04-22

**Authors:** Steven R. Talbot, Miriam Heider, Martin Wirth, Anne Jörns, Ortwin Naujok

**Affiliations:** 1 Institute of Clinical Biochemistry, Hannover Medical School, 30625 Hannover, Germany; 2 Hannover Medical School, Institute for Laboratory Animal Science, Carl-Neuberg-Straβe 1, 30625 Hannover, Germany

Correction to: *Scientific Reports* 10.1038/s41598-024-67490-z, published online 17 July 2024

The original version of this Article contained an error in Affiliation 2, which was incorrectly given as ‘Institute for Laboratory Animal Science, Hannover, Germany.’ The correct affiliation is listed below.

Hannover Medical School, Institute for Laboratory Animal Science, Carl-Neuberg-Straβe 1, 30625 Hannover, Germany.

The original Article has been corrected.

