# Supplementary material for: Exploring dose–response variability and relative severity assessment in STZ-induced diabetes male NSG mice
Source: Sci Rep. 2024 Jul 17;14:16559. doi: 10.1038/s41598-024-67490-z (PMC11255292; doi:10.1038/s41598-024-67490-z)
Supplement: Supplementary file 1 — Supplementary Information. [file 41598_2024_67490_MOESM1_ESM.docx]

**Glucose and body weight analysis**

A mixed-effects analysis of variance (ANOVA) was used to estimate the effect of treatment levels (doses) and time (day) on glucose/body weight levels. Glucose/body weight was the dependent variable, and day and treatment were the independent variables. To assess whether the effect of treatment varied over time, an interaction effect of 'treatment:day' was included in the model. Additionally, we accounted for the within-subjects correlation using a hierarchical error matrix. This structure indicated that the repeated measurements for subjects (id) were nested within the combinations of treatment and day. These random effects helped explain variability for the within-subjects differences separately from the main and interaction effects. The residual errors of the model were inspected after the fit to ensure that ANOVA assumptions were met.

**Table 1.** Hierarchical analysis of blood glucose levels over time.

| **Error: id** |  |  |  |  |  |
| --- | --- | --- | --- | --- | --- |
|  | **Df** | **SS** | **MSQ** | **F** | **p** |
| **dose** | 5 | 9983 | 1996.6 | 122.5 | <0.0001 |
| **day** | 8 | 1457 | 182.1 | 11.2 | <0.0001 |
| **responder** | 1 | 829 | 829.3 | 50.9 | <0.0001 |
| **dose:day** | 4 | 248 | 62.1 | 3.8 | 0.011 |
| **dose:responder** | 1 | 7 | 7.1 | 0.4 | 0.512 |
| **Residuals** | 38 | 620 | 16.3 |  |  |
|  |  |  |  |  |  |
| **Error: id:day** |  |  |  |  |  |
|  | **Df** | **SS** | **MSQ** | **F** | **p** |
| **dose:day** | 38 | 4746 | 124.9 | 15.9 | <0.0001 |
| **day:responder** | 11 | 1265 | 115 | 14.6 | <0.0001 |
| **dose:day:responder** | 14 | 114 | 8.1 | 1 | 0.42 |
| **Residuals** | 337 | 2648 | 7.9 |  |  |

**Table 2.** Hierarchical analysis of body weight change over time.

|  | **Df** | **SS** | **MSQ** | **F** | **p** |
| --- | --- | --- | --- | --- | --- |
| **dose** | 5 | 6435 | 1287.1 | 41.9 | <0.0001 |
| **day** | 7 | 959 | 137.1 | 4.5 | <0.001 |
| **responder** | 1 | 0 | 0.2 | 0.005 | 0.942 |
| **dose:day** | 3 | 281 | 93.7 | 3.1 | 0.039 |
| **dose:responder** | 1 | 4 | 4.4 | 0.14 | 0.706 |
| **Residuals** | 40 | 1229 | 30.7 |  |  |
|  |  |  |  |  |  |
| **Error: id:day** |  |  |  |  |  |
|  | **Df** | **SS** | **MSQ** | **F** | **p** |
| **dose:day** | 38 | 1896.8 | 49.92 | 10 | <0.0001 |
| **day:responder** | 10 | 60.4 | 6.04 | 1 | 0.274 |
| **dose:day:responder** | 17 | 117.2 | 6.9 | 1 | 0.134 |
| **Residuals** | 316 | 1557.3 | 4.93 |  |  |

**Supplementary Table 3: ANOVA table of the regression for RELSA**

|  | **Df** | **Sum Sq** | **Mean Sq** | **F-value** | **Pr(>F)** |
| --- | --- | --- | --- | --- | --- |
| **Treatment** | 5 | 7.420 | 1.4841 | 39,16 | <2e-16 *** |
| **Residuals** | 52 | 1.971 | 0.0379 |  |  |

Significance codes: 0 ‘***’ 0.001 ‘**’ 0.01 ‘*’

**Supplementary Table 4: Body scoring system used in the study**

| **Score** | **Activity** | **Body weight** | **General condition** | **Behavior** | **Measures** |
| --- | --- | --- | --- | --- | --- |
| **1** | Very active | +/- 5 % | Fur smooth and shiny; eyes clear and shiny;  Body orifices clean | lively, attentive, curious, movements typical of the species | checks:  Day -1 to day 4: daily  from day 5 on: 3x per week |
| **2** | active | 5-10 % | Fur smooth and shiny; eyes clear and shiny;  Body orifices clean | lively, movements typical of the species, signs of PU/PD, polydipsia  Blood glucose  > 7 and < 15 mmol/l | Daily checks |
| **3** | calm, less active | 10-15 % | Fur smooth and shiny; eyes clear but almond-shaped shiny;  Body orifices clean | alert, somewhat calmer, reduced movement, posture typical of the species, clear signs of PU/PD, blood glucose  >15 mmol/l | twice daily checks  soaked food on the cage floor  if necessary, additional measurement of blood glucose  Experiment stopped after 96 hours in diabetic animals  Immediate termination if BGW >30 mml/l in two consecutive measurements |
| **4** | Limited activity | ≤ 20 % | Fur dull, erect; eyes no longer fully open; body orifices untended | Animal calm, frequent lingering, slightly curved back line, reduced personal hygiene, limited reactions to environmental stimuli, blood glucose  >15 mmol/l | Immediate termination of the experiment as soon as one criterion from score 4 is recorded |
| **5** | No activity, lethargic | > 20 % | Fur erect, dirty; eyes closed; body orifices dirty or moist | self-isolation; strongly curved back line, no significant activity, hardly any reaction to environmental stimuli | Immediate termination |

PU = polyuria, PD = polydipsia
